# Identification and Characterization of MicroRNAs in Asiatic Cotton (*Gossypium arboreum* L.)

**DOI:** 10.1371/journal.pone.0033696

**Published:** 2012-04-06

**Authors:** Min Wang, Qinglian Wang, Baomin Wang

**Affiliations:** 1 College of Agronomy and Biotechnology, China Agricultural University, Beijing, China; 2 Henan Institute of Sciences and Technology, Xingxiang, Henan, China; East Carolina University, United States of America

## Abstract

To date, no miRNAs have been identified in the important diploid cotton species although there are several reports on miRNAs in upland cotton. In this study, we identified 73 miRNAs, belonging to 49 families, from Asiatic cotton using a well-developed comparative genome-based homologue search. Several of the predicted miRNAs were validated using quantitative real time PCR (qRT-PCR). The length of miRNAs varied from 18 to 22 nt with an average of 20 nt. The length of miRNA precursors also varied from 46 to 684 nt with an average of 138 ±120 nt. For a majority of Asiatic cotton miRNAs, there is only one member per family; however, multiple members were identified for miRNA 156, 414, 837, 838, 1044, 1533, 2902, 2868, 5021 and 5142 families. Nucleotides A and U were dominant, accounted for 62.95%, in the Asiatic cotton pre-miRNAs. The Asiatic cotton pre-miRNAs had high negative minimal folding free energy (MFE) and adjusted MFE (AMFE) and high MFE index (MFEI). Many miRNAs identified in Asiatic cotton suggest that miRNAs also play a similar regulatory mechanism in diploid cotton.

## Introduction

Cotton is an important economic crop due to its role in providing a source of fiber, textile, and oils. Besides the fact that cotton is the most important fiber crop; cotton is also the second most important resource for plant protein and the fifth most important oil crop. Cotton seeds contain about 23% proteins, and it is estimated that cotton seeds could potentially provide the protein required by about half billion people annually. Because cotton seeds are rich in oil content (approximately 21%), it has become the fifth most important plant oil resource in the world.. Although cotton is an important crop, scientific research and genetic knowledge of cotton is much behind other crops, such as rice, maize, wheat and soybean.

The genus *Gossypium* is a large family, which consists of approximately 50 species originating from different geographic areas, majorly from Australia and Latin America. However, only four of them are domesticated and produce valuable spinnable fiber. The four domesticated species are Upland cotton (*Gossypium hirsutum* L.), Island cotton (*G. barbadense* L.), African cotton (*G. herbaceum* L.) and Asiatic cotton (*G. arboreum* L.). The Upland cotton and Island cotton are allotetraploids (AADD, 2n  =  4x  =  52); African cotton (A1A1) and Asiatic cotton (A2A2) are diploids (2n  =  2x  =  26). Because tetraploid cotton (Upland Island cotton) have much higher fiber yield as well longer fibers than the diploid cotton ( African and Asiatic cotton), tetraploid cotton, particularly the Upland cotton, is currently widely cultivated around the world. Despite their poor yield and fiber quality, the diploid species have many other attractive agronomic traits [1]. For example, several investigations demonstrated that the Asiatic cotton was tolerant and even resistant to several environmental biotic stresses, such as resistance to several pests and diseases, including bollworms [2], aphids [3], leafhopper [3], rust, fungal [4], and viral [5] diseases. Asiatic cotton also contains traits tolerant to environmental abiotic stress, including drought and salinity stress [6], [7]. Thus, Asiatic cotton easily grows in dry land with low input cultivation practices. Many Asiatic cotton cultivars produce fibers with high strength and some produc seeds with high oil content. All of these traits make Asiatic cotton a valuable resource of genetic germplasm or gene pool for modern cotton cultivar improvement and the cotton industry [1]. Additionally, diploid cotton also has an advantage over tetraploid cotton in elucidating gene structure and function because their genome is much more simple as compared to the tetraploid species. Unfortunately, very few studies have focused on diploid cotton, particularly on the molecular level. Understandinggene function and their regulatory mechanisms could provide new insight into the mechanisms of cotton growth and development, particularly on cotton fiber initiation and development and the response to environmental abiotic and biotic stresses. The expected results of this study will facilitate efficient usage of the diploid cotton species for developing improved cotton cultivars with favorable fiber yield and other key agronomic traits.

microRNAs (miRNAs) are a newly identified class of extensively endogenous small RNA molecules and are approximately 20–22 nucleotides in length. Increasing amounts of evidence shows that miRNAs play an essential role in almost all biological and metabolic processes, such as plant organ differentiation and development, signal transduction, phase change, and response to environmental biotic and abiotic stress [8]. For example, miR 172 controls flower development and phase change from vegetative growth to reproductive growth though targeting the apetala 2 gene [9], [10], [11], [12]. miRNAs are different from other RNAs, including small interfering RNAs (siRNAs). The important features of miRNAs include the following: 1) all miRNAs are coded by miRNA genes with unknown length; a miRNA gene is first transcribed into a long primary RNAs, called primary miRNA (pri-miRNAs). A pri-miRNA is sequentially cleaved into a short stem-loop structured miRNA precursor (pre-miRNA) and then to a mature miRNA by an enzyme called dicer-like 1 (*dcl 1*) with the help of other several other enzymes. 2) All pre-miRNAs can form a stem-looped hairpin secondary structure with the mature miRNA siting on one of the arms. 3) A pre-miRNA forms its secondary structure with high negative minimum folding free energy (MFE) and minimum folding free energy index (MFEI). 4) All miRNAs have their star sequence, the opposite site sequence, termed miRNA*, in the secondary hairpin structure; miRNA and miRNA* sequences are almost complementary to each other with few mismatches; therefore miRNA and miRNA* sequences always form miRNA:miRNA* complexes during miRNA biogenesis. In most cases, only miRNAs function in regulating gene expression and the miRNA* sequence is degraded by an unknown mechanism. However, in some cases, the miRNA* sequence also can function to target a specific gene.

More interestingly, many miRNAs are highly evolutionarily conserved from species to species in both animal and plant kingdoms although no clue for that miRNAs were a common ancestor for plant and animal miRNAs. This conservation ranges a long distance, such as from worms to humans in animals [13], [14], [15] and from moss and ferns to high flowering plants in plants [16]. This provides a powerful strategy to identify miRNAs in a new plant species using already known miRNAs in a known plant species through the use of a comparative genome-based homologue search. However, conservation is not the only criteria used for identifying miRNAs using a homologue-based Blastn search because miRNAs are short and it is difficult to distinguish a miRNA sequence from their targeted gene sequences. When using homologue-based Blastn searches for miRNAs, other criteria, such as their secondary hairpin stem-loop structure must also be considered. Since comparative genome-based homologue searcesh were employed to identify plant miRNAs in the middle of 2000s [17], this strategy has been widely adopted by many research laboratories [18]. Based on a rough approximation, using this method, thousands of miRNAs have been systematically identified in more than 30 plant species, including many important plant and agricultural crop species, such as soybean [19], [20], maize [21], tomato [22], tobacco [23], potato [24], [25], wheat [26], *Brassica napus* [27], citrus [28], switchgrass [29] and apple [30].

According to miRBase, a public miRNA database (Release 18, November 2011), there are a total of 4,014 plant miRNAs that have been currently identified and deposited into this database [31]. These miRNAs are obtained from 52 plant species, ranging from ferns and mosses to high flowering plants. However, no study has been reported in Asiatic cotton, one of the most important cotton species. In this study, we employed a comparative genome-based homologue search to identify miRNAs in Asiatic cotton using current Asiatic cotton expressed sequence tags (ESTs) available in the NCBI Genbank database. Based on our findings, 73 miRNAs, belonging to 49 families have been identified for the first time in Asiatic cotton. A majority of these miRNAs also play important roles in other plant species. We will also report the major features of these newly identified Asiatic cotton miRNAs.

## Materials and Methods

### Reference Set of miRNAs

Currently, a total of 4,014 miRNAs from 52 plant species have been deposited in miRBase, a public available miRNA database. These miRNAs are defined as a reference set of miRNA sequences for identifying potential miRNAs in Asiatic cotton. All currently available mature miRNAs and their precursor sequences were downloaded from miRBase (http://www.mirbase.org/; Release 18, November 2011). After the miRNA sequences were downloaded, mature miRNA sequences were Blasted against each other to remove identical sequences. Only unique sequences were kept for later Blastn seaches for identifying Asiatic cotton miRNAs.

### Asiatic Cotton ESTs, cDNAs, and mRNAs

Asiatic cotton expressed sequence tags (ESTs), cDNAs, and mRNAs were downloaded from the GenBank nucleotide databases at the National Center for Biotechnology Information (NCBI). Currently, a total of 41,781 Asiatic cotton ESTs are available in the NCBI EST database (dbEST release 120111, December 1, 2011; http://www.ncbi.nlm.nih.gov/dbEST/dbEST_summary.html).

### Identifying Potential Asiatic Cotton miRNAs Using a Comparative Genome-based Homologue Search and Secondary Structure Analysis

A comparative genome-based homologue search is a powerful approach for identifying conserved miRNAs in both plants and animals. Since it was developed in 2005, this method has been widely employed to identify miRNAs in various plant species and also in animals. In this study, we employed the previously published method to identify Asiatic cotton miRNAs from currently available EST sequences. A comparative genome-based homologue search is based on two key parameters: conservation of miRNA sequences and the second stem-loop hairpin structure of the potential pre-miRNAs. Both factors should be strictly considered during identifying conserved miRNAs. Because only mature miRNAs are highly evolutionary conserved in plants for the majority of miRNAs, only mature miRNA sequences were used for the Blast search in this study. The mature sequences of all currently available plant miRNAs were subjected to a Blastn search against all of the currently available Asiatic cotton EST sequences. To identify all potential Asiatic cotton miRNAs, the following Blast parameters were used according to the previous studies: 1) the default word-match size was set at seven, the smallest setting that can be used for the online Blastn program between the miRNA query and the EST sequences; 2) the expected values were set at 1,000 to increase the hit chance for more potential sequences; and 3) the sequence number of the Blastn search and the sequence alignments were set to 1,000. More likely, for the majority of the Blastn search, only partial miRNA sequences were matched to the EST sequences. If this occured, the non-aligned regions of the miRNA query were manually inspected and aligned to the hit EST sequence; the number of mismatched nucleotides was recorded for determining whether the EST sequences contained a potential miRNA sequence according to the criteria described in the later section.

All ESTs with no more than 3 mismatches were selected for further investigation. First, the selected ESTs were Blasted against each other to remove the repeated sequences. Then, the secondary structures of the remaining EST sequences were predicted using a web-based computational program, mfold (http://mfold.rna.albany.edu/?q=mfold/RNA-Folding-Form). All mfold outputs were recorded into an excel file, which included the number of each nucleotide (A, G, C and U), the number of arms per structure, location of the matching regions, and minimal folding free energy (MFE, ΔG kcal/mol). Then, the adjust minimal folding free energy (AMFE) and the minimal folding free energy index (MFEI) were calculated according to the previous report [32]. AMFE means the MFE of a RNA sequence with 100 nt in length, which is equal to MFE/(length of a potential pre-miRNA) * 100. MFEI is equal to MFE/(length of a potential pre-miRNA)/(The percentage of nucleotides G and C) [32].

An EST was considered a miRNA candidate when it fit all of the following criteria: 1) the predicted mature miRNAs had no more than three nucleotide substitutions compared with a known mature miRNAs; 2) the EST sequence could fold into an appropriate stem-loop hairpin secondary structure; 3) the mature miRNA was localized in one arm of the stem-loop structure; 4) there was no loop or break in the miRNA or miRNA* sequences; 5) there were no more than 6 mismatches between the predicted mature miRNA sequence and its opposite miRNA* sequence in the secondary structure; and 6) the predicted secondary structure had high negative MFE and high MFEI value.

### Validation of Asiatic Cotton miRNAs

The predicted Asiatic cotton miRNAs were validated using stem-loop RT-PCR according to the previous report [33]. Total RNA was isolated from 10-day-old seedlings of Asiatic cotton following a previous report. After measuring the concentraion and quality of the RNAs, 200 ng of total RNA was used to reverse transcribe a miRNA into cDNA using a miRNA-specific stem-loop primer and the TaqMan® microRNA Reverse Transcription kit (Applied Biosystems) according to the manufacturer’s protocol.

Quantitative real time PCR (qRT-PCR) was performed on an Applied Biosystems 7300 Sequence Detection System. For each reaction, 20 µL PCR reaction mixtures were prepared and each contained 2 µL of RT product from the reverse-transcription reaction (after tenfold dilution), 2 µL of miRNA-specific primers, 10 µL Universal Syber Green PCR Master Mix, and 6 µL nuclease-free water. The reactions were incubated in a 96-well plate at 95°C for 10 min, followed by 45 cycles of 95°C for 15 s and 60°C for 60 s. After the completion of the real-time reactions, the threshold was manually set and the threshold cycle (C_T_) was recorded. All reactions were conducted in triplicate.

## Results

### Identification of miRNAs in Asiatic Cotton

**Figure 1:**
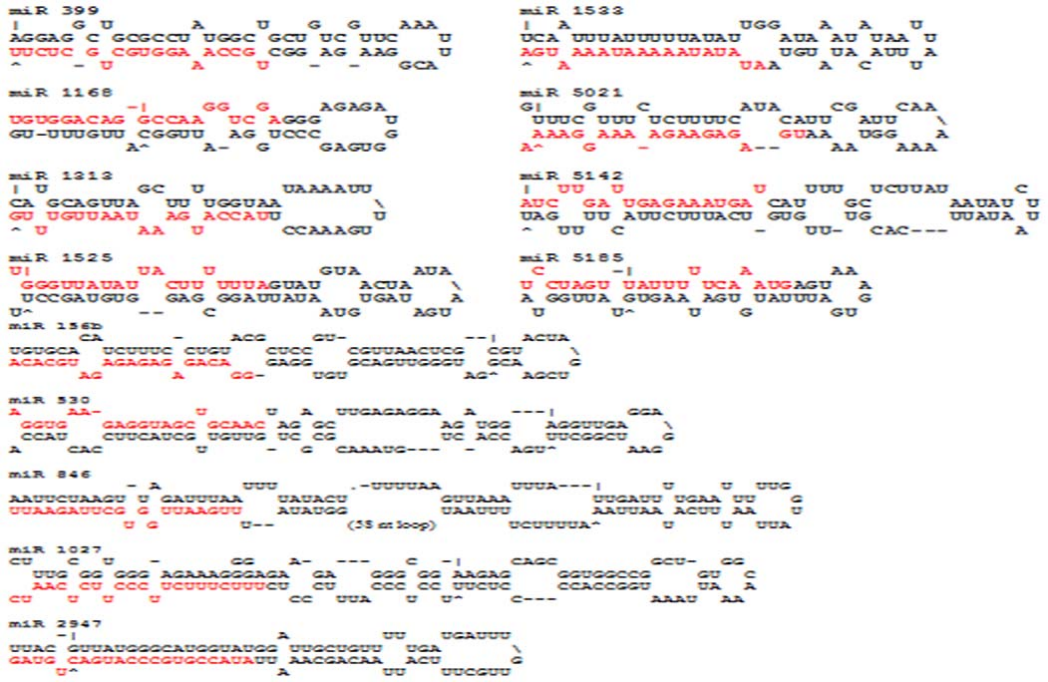
Predicted stem-loop hairpin secondary structures of the selected Asiatic cotton miRNAs identified in this study. Mature miRNA sequences are in red color. The length of the accurate miRNA precursors may be slightly longer than what is presented here.

After carefully considering the Blastn results and the secondary structure of the sequences, we identified 73 potential miRNAs from the 41,781 currently available Asiatic cotton ESTs. These 73 miRNAs belong to 49 families (Table 1, Figure 1 and Table S1). A large number of miRNAs identified in Asiatic cotton suggests that miRNAs also widely exist in diploid cotton species and play important roles in Asiatic cotton growth and development based on the evidences in other plant species.

**Table 1:** Asiatic cotton miRNAs identified by homolog search and secondary structure.

Family	miRNA	Mature Sequence	ML	Lc	PL	A	C	G	U	AT	GC	A/U	C/G	MFE	AMFE	MFEI
156	156a	CGACAGAAGCAAGAGGCAC	19	3	119	26.89	26.89	28.57	17.65	44.54	55.46	1.52	0.94	35.3	29.66	0.535
	156b	GGACAGAGAGAGAGATGCACA	21	3	92	22.83	21.74	31.52	23.91	46.74	53.26	0.95	0.69	39.6	43.04	0.808
	156c	TTACAGAAGAGAAGATCAC	19	5	82	36.59	15.85	20.73	26.83	63.41	36.59	1.36	0.76	19.9	24.27	0.663
	156d	AGAGAGAGATAGAGAGACAG	20	5	52	32.69	13.46	23.08	30.77	63.46	36.54	1.06	0.58	16.6	31.92	0.874
158	158	TCCCAAATCTGAGACAAAGA	20	5	57	26.32	12.28	29.82	31.58	57.89	42.11	0.83	0.41	16.6	29.12	0.692
167	167	TGAAGCTGCATGCATGATGTC	21	5	65	24.62	24.62	20.00	30.77	55.38	44.62	0.80	1.23	14	21.54	0.483
172	172	TGAACCTGATGCTGCTGCAG	20	5	52	19.23	21.15	38.46	21.15	40.38	59.62	0.91	0.55	22.9	44.04	0.739
399	399	TGCCAAAGGTGCTGCTCTT	19	3	65	20.00	23.08	32.31	24.62	44.62	55.38	0.81	0.71	27.4	42.15	0.761
414	414a	TGATCATCATCATCATCGTCG	21	5	386	23.58	18.65	38.86	18.91	42.49	57.51	1.25	0.48	126.9	32.88	0.572
	414b	TCATCATCATCATCATCTTCA	21	5	373	25.20	21.72	15.82	37.27	62.47	37.53	0.68	1.37	86.6	23.22	0.619
482	482	CCTTGCCTCTCCTCCCTTT	19	5	180	36.67	16.11	15.56	31.67	68.33	31.67	1.16	1.04	38.7	21.50	0.679
530	530	AGGTGAAGAGGTAGCTGCAAC	21	5	104	26.92	18.27	31.73	23.08	50.00	50.00	1.17	0.58	34.3	32.98	0.660
773	773	CTTGATTGCAGCTTTTGTTTC	21	3	53	20.75	13.21	30.19	35.85	56.60	43.40	0.58	0.44	7.9	14.91	0.343
780	780	TATACCAGCTTTTGAGCAGGT	21	3	48	22.92	18.75	27.08	31.25	54.17	45.83	0.73	0.69	18.5	38.54	0.841
821	821	AATTAATAAAATAAAAGTTG	20	3	52	36.54	0.00	5.77	57.69	94.23	5.77	0.63	0.00	4.5	8.65	1.500
837	837a	CATTGTTTCTTCTTCTTTTCT	21	3	115	29.57	20.87	13.91	35.65	65.22	34.78	0.83	1.50	26.1	22.70	0.653
	837b	CTTTGTTACTTGTATTTTTCA	21	5	47	38.30	10.64	14.89	36.17	74.47	25.53	1.06	0.71	6.9	14.68	0.575
838	838a	TTTTCTTCTCCTTCTTTACA	20	3	89	25.84	22.47	20.22	31.46	57.30	42.70	0.82	1.11	27.2	30.56	0.716
	838b	TTTTCTTTTCTCTTCCACA	19	3	55	36.36	16.36	14.55	32.73	69.09	30.91	1.11	1.13	13.5	24.55	0.794
840	840	ACACTAAAGACAAACTAAC	19	5	470	28.51	23.83	14.47	33.19	61.70	38.30	0.86	1.65	101	21.49	0.561
846	846	TTGAATTGGTGCTTAGAATT	20	3	178	28.65	5.06	12.92	53.37	82.02	17.98	0.54	0.39	32.9	18.48	1.028
851	851	TGGATGGCAACAAAGACGAT	20	5	120	27.50	26.67	24.17	21.67	49.17	50.83	1.27	1.10	34.4	28.67	0.564
902	902	TATGACGCAGCTTCTTAA	18	5	209	33.01	21.05	13.88	32.06	65.07	34.93	1.03	1.52	34.5	16.51	0.473
952	952	ATCTGAGAAGCCATCTGGTG	20	5	337	25.82	23.74	25.82	24.63	50.45	49.55	1.05	0.92	113.7	33.74	0.681
1027	1027	TTTCTTTCTTCCCTTCTCAATC	22	3	118	19.49	27.97	26.27	26.27	45.76	54.24	0.74	1.06	48.3	40.93	0.755
1044	1044a	TTGAGTGCTTATTTGTCTT	19	5	65	23.08	16.92	20.00	40.00	63.08	36.92	0.58	0.85	14.6	22.46	0.608
	1044b	TTGTAGTGAATATGTGATTT	20	3	46	30.43	10.87	23.91	34.78	65.22	34.78	0.88	0.45	6.6	14.35	0.413
1092	1092	TGAAGGAAGAATTGGTGTT	19	3	217	35.94	13.36	19.82	30.88	66.82	33.18	1.16	0.67	46.8	21.57	0.650
1104	1104	CGCTGCTGTTTTTGTTTCCTTC	22	5	150	11.33	18.00	43.33	27.33	38.67	61.33	0.41	0.42	58.9	39.27	0.640
1168	1168	TGTGGACAGGCCAAGGTCGA	20	5	58	20.69	13.79	39.66	25.86	46.55	53.45	0.80	0.35	19.3	33.28	0.623
1313	1313	TACCATGAAATAATTGTTTG	20	3	57	33.33	12.28	15.79	38.60	71.93	28.07	0.86	0.78	10.6	18.60	0.663
1525	1525	TGGGTTATATTACTTTTTTAGT	22	5	67	28.36	7.46	22.39	41.79	70.15	29.85	0.68	0.33	17.4	25.97	0.870
1530	1530	TTTACAAATAAATTAAATAT	20	5	345	35.65	6.96	13.33	44.06	79.71	20.29	0.81	0.52	59.8	17.33	0.854
1533	1533a	ATATATAAAAGTAATAATGC	20	5	165	32.12	10.91	7.88	49.09	81.21	18.79	0.65	1.38	22	13.33	0.710
	1533b	ATAACAATAATAGTAATGA	19	3	46	36.96	13.04	13.04	36.96	73.91	26.09	1.00	1.00	5.1	11.09	0.425
	1533c	ATATATAAAAATAAAATGA	19	3	64	46.88	3.13	6.25	43.75	90.63	9.38	1.07	0.50	11.1	17.34	1.850
	1533d	CTAATAATAATAGTAATGA	19	5	52	30.77	5.77	15.38	48.08	78.85	21.15	0.64	0.38	3.2	6.15	0.291
	1533e	AGAATAAAAATAATAATGT	19	3	66	34.85	4.55	24.24	36.36	71.21	28.79	0.96	0.19	12.5	18.94	0.658
1535	1535	CTTGGTTTTTGGTGATTCT	19	3	137	31.39	12.41	24.82	31.39	62.77	37.23	1.00	0.50	34.1	24.89	0.669
1846	1846	TTTCCGGCGCCCCAAGGAGG	20	5	76	30.26	23.68	25.00	21.05	51.32	48.68	1.44	0.95	19.9	26.18	0.538
1888	1888	TAAGTTTGATTTGTGAAGTA	20	5	174	25.86	14.94	24.14	35.06	60.92	39.08	0.74	0.62	47.4	27.24	0.697
2079	2079	GGTGTTGATGTGATGACGCA	20	3	64	26.56	21.88	21.88	29.69	56.25	43.75	0.89	1.00	18.5	28.91	0.661
2092	2092a	GAACTGAAGTGGGTTTTTACT	21	3	168	30.95	8.33	26.19	34.52	65.48	34.52	0.90	0.32	37.5	22.32	0.647
	2092b	CAATGAAGTAGGTGTGTACT	20	3	49	32.65	10.20	24.49	32.65	65.31	34.69	1.00	0.42	10.5	21.43	0.618
2093	2093	ACATTTTCCAATTAATGCTT	20	3	50	32.00	14.00	18.00	36.00	68.00	32.00	0.89	0.78	12.4	24.80	0.775
2095	2095	CTTCCATGTAGGATAATTAT	20	5	81	25.93	22.22	19.75	32.10	58.02	41.98	0.81	1.13	14	17.28	0.412
2108	2108	TTATATGTGTGTGTTTTGAG	20	3	68	26.47	16.18	25.00	32.35	58.82	41.18	0.82	0.65	17.8	26.18	0.636
2275	2275	AGTGTTGAGGAAAGAAACC	19	3	374	34.49	13.64	16.04	35.83	70.32	29.68	0.96	0.85	75	20.05	0.676
2607	2607	ATGTATTTTGTGATAGTGT	19	3	104	28.85	17.31	15.38	38.46	67.31	32.69	0.75	1.13	23.2	22.31	0.682
2630	2630	TGGTTTTGGTCTTTGATATTT	21	3	129	26.36	10.85	26.36	36.43	62.79	37.21	0.72	0.41	33.6	26.05	0.700
2868	2868a	TTGGTTTTTGAAGGAGAAA	19	3	156	35.90	17.31	14.74	32.05	67.95	32.05	1.12	1.17	27.6	17.69	0.552
	2868b	TTGTTTTTGTGTGTAGAAG	19	3	163	30.06	12.88	21.47	35.58	65.64	34.36	0.84	0.60	41.5	25.46	0.741
2911	2911	GGGCGGGGGAGGGGCTGAGGA	21	3	684	19.15	15.50	45.18	20.18	39.33	60.67	0.95	0.34	232.2	33.95	0.560
2947	2947	TATACCGTGCCCATGACTGTAG	22	3	86	22.09	15.12	23.26	39.53	61.63	38.37	0.56	0.65	44.6	51.86	1.352
3447	3447	TTTGAGTTGTAAGTTATGAA	20	3	50	36.00	4.00	28.00	32.00	68.00	32.00	1.13	0.14	6.7	13.40	0.419
4248	4248	GTATTTTATTTTTTGGCAATCA	22	5	69	24.64	8.70	27.54	39.13	63.77	36.23	0.63	0.32	11.5	16.67	0.460
4413	4413	AAGAGTATGTAAGCACTG	18	5	51	25.49	17.65	21.57	35.29	60.78	39.22	0.72	0.82	13.2	25.88	0.660
5020	5020	ATGGTGAAGAAGGTGAGA	18	3	191	28.27	17.28	28.27	26.18	54.45	45.55	1.08	0.61	63.3	33.14	0.728
5021	5021a	AGAGAAGAAGAAGAGAAAA	19	5	380	33.42	27.37	17.37	21.84	55.26	44.74	1.53	1.58	100	26.32	0.588
	5021b	TGATAAAAGAAGAAGAAAA	19	5	192	23.96	21.88	19.27	34.90	58.85	41.15	0.69	1.14	56.4	29.38	0.714
	5021c	AGAGAAGAAGACGAAGAAGA	20	5	207	33.33	21.26	20.77	24.64	57.97	42.03	1.35	1.02	59.5	28.74	0.684
	5021d	TGAGAAGAAAAAGAAGAAAG	20	5	176	36.93	11.93	23.30	27.84	64.77	35.23	1.33	0.51	48.4	27.50	0.781
	5021e	TGAGAGAAGAAAAGGAAAA	19	3	62	41.94	11.29	17.74	29.03	70.97	29.03	1.44	0.64	10.6	17.10	0.589
	5021f	TAAGAAAGAAAAAGAAGAAAA	21	3	354	49.72	7.63	9.32	33.33	83.05	16.95	1.49	0.82	51.6	14.58	0.860
	5021g	TGAAAAGAAGAAAAGAAAG	19	3	167	40.12	7.78	20.36	31.74	71.86	28.14	1.26	0.38	34.3	20.54	0.730
	5021h	TTAGAAGAAGATGAAGACAA	20	5	146	24.66	24.66	22.60	28.08	52.74	47.26	0.88	1.09	45.3	31.03	0.657
	5021i	GGAGAATATGAAGAAGAAAA	20	3	49	42.86	4.08	16.33	36.73	79.59	20.41	1.17	0.25	11.3	23.06	1.130
	5021j	TAAGAAGAAGAAGAAGAATA	20	5	55	34.55	18.18	14.55	32.73	67.27	32.73	1.06	1.25	25.7	46.73	1.428
5053	5053	CGCCGCTGTCGTCGCCGCCGT	21	3	149	21.48	30.20	20.81	27.52	48.99	51.01	0.78	1.45	48.1	32.28	0.633
5142	5142a	ACATTGATTATAACTGAT	18	5	72	40.28	11.11	15.28	33.33	73.61	26.39	1.21	0.73	6.6	9.17	0.347
	5142b	ATATGCTTGATATGTGAT	18	3	129	32.56	9.30	20.16	37.98	70.54	29.46	0.86	0.46	28	21.71	0.737
	5142c	ATCTTGATTGAGAAATGAT	19	5	75	25.33	13.33	12.00	49.33	74.67	25.33	0.51	1.11	13	17.33	0.684
5185	5185	TCCTAGTTATTTTTCAAATG	20	5	53	30.19	5.66	20.75	43.40	73.58	26.42	0.70	0.27	9.3	17.55	0.664

ML: mature sequence length; Lc: the location of arm; PL: the length of pre-miRNAs.

Although miRNA mature sequences are highly evolutionarily conserved from species to species, in this study, we found that a majority of identified mature Asiatic cotton miRNAs had 2 or 3 nucleotide changes as compared with miRNAs in other plant species. This may be caused by two reasons: 1) due to the limited EST sequences available in the database, we only identified partial miRNAs identified in other plant species. For many high evolutionally conserved miRNAs, such as miR 162 and miR 164, we did not find those in this study. As more EST sequences become available in the database, it is possible that we could identify more miRNAs that are identical with currently known miRNAs. 2) Although miRNAs are highly conserved, it does not mean the miRNAs are identical among different species and there may be mutantions in the evolution of miRNA biogenesis.

### Characterization of miRNAs in Asiatic Cotton

The identified miRNAs were not evenly distributed in each miRNA family (Table 1). For a majority of the miRNA families identified, there was only one member identified in this study. However, for some miRNA families (miRNA 156, 414, 837, 838, 1044, 1533, 2902, 2868, 5021 and 5142), multiple members were identified. For example, there are four members identified in the miR156 family. Although this may be caused by the uneven EST sequences, it also suggests the potential miRNA patterns in Asiatic cotton. miR156 is a large family in other plant species, such as the model plant species *Arabidopsis*, which plays an important role in leaf development.

A majority of identified miRNAs were obtained from the plus strand. However, there were several miRNAs identified from the minus strand of certain ESTs. The mature miRNA sequences could be located within either the 3' or 5' arm of the secondary stem-loop hairpin structures. Among the 73 identified miRNAs, 38 were located within the 3' arm and the other 35 miRNAs were located with the 5' arm.

**Figure 2:**
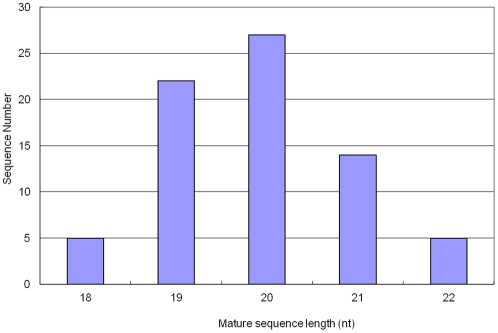
The length distribution of mature miRNAs identified in Asiatic cotton . The range of length is 18–22 nt with an average of 19.9±1.0 nt.

The length of mature miRNAs varied from 18 to 22 nt with an average of 19.9±1.0 nucleotides (Figure 2). A majority of mature miRNAs have 19–21 nt, which counts for 86.30% of the total identified miRNAs. The length range of the Asiatic cotton mature miRNAs is similar to those of other plant species.

**Figure 3:**
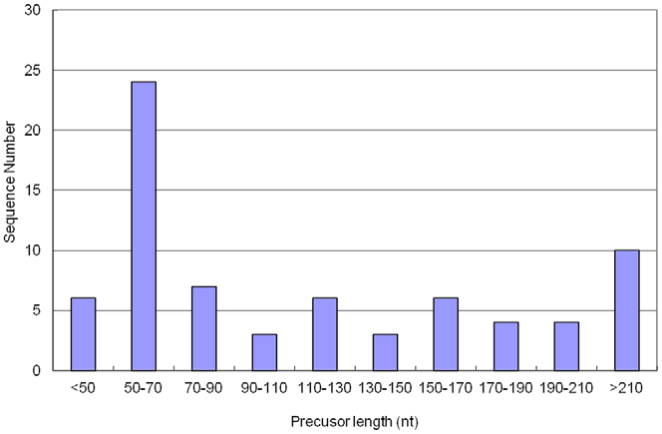
The length distribution of pre-miRNAs identified in Asiatic cotton. The range of length is 46–684 nt with an average of 138±120 nt.

The length of identified miRNA precursor sequences was also varied from miRNA to miRNA. The range of pre-miRNA lengths is 46 to 684 nt with an average of 138±120 nt (Figure 3). However, a large percentage of pre-miRNAs (32.9%) had 50–70 nt. There were only 10 miRNAs (13.7%) with more than 210 nt in length.

**Figure 4:**
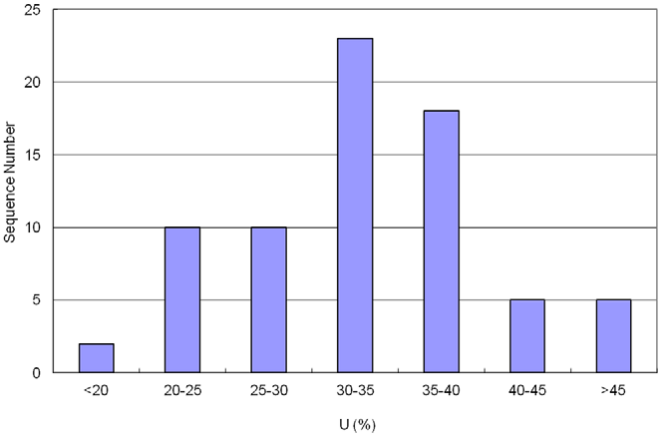
The distribution of U content. The range is 17.65–57.69% with an average of 33.09±7.85%.

The composition of the four nucleotides (A, G, C, and U) is an important parameter, which is an indicator for species evolution as well as for the stabilization of one specific RNA sequence cased by their secondary structure. The percentage composition of each nucleotide was not evenly distributed in the identified Asiatic cotton pre-miRNAs (Table 2). With an unknown reason, the nucleotide uracil (U) is dominant in both mature miRNAs and pre-miRNAs in both plant and animal miRNAs. In this study, we observed that the U content varied from 17.7% to 57.7% with an average of 33.1±7.9% in the identified Asiatic cotton pre-miRNAs (Figure 4); which is significantly higher than the content of other nucleotides, particularly much higher than nucleotides C (15.4±6.8%) and G (21.7±8.0%). A majority (83.7%) of pre-miRNAs contained more than 25% of the nucleotide U (Figure 4).

**Table 2:** Statistics of the characterized parameters of Asiatic cotton miRNA precursors.

Parameter	Mean	Standard Deviation	Minimal	Maximal
MFE (▵G, -kcal/mol)	35.19	35.12	3.2	232.2
AMFE( -kcal/mol)	24.92	9.21	6.15	51.86
MFEI	0.70	0.25	0.29	1.85
Length (nt)	138	120	46	684
(G+C)%	37.05	11.78	5.77	61.33
(U+A)%	62.95	11.78	38.67	94.23
A%	29.86	6.91	11.33	49.72
C%	15.36	6.81	0.00	30.20
G%	21.69	7.99	5.77	45.18
U%	33.09	7.85	17.65	57.69
A/U ratio	0.94	0.26	0.41	1.53
C/G ratio	0.76	0.38	0.00	1.65

**Figure 5:**
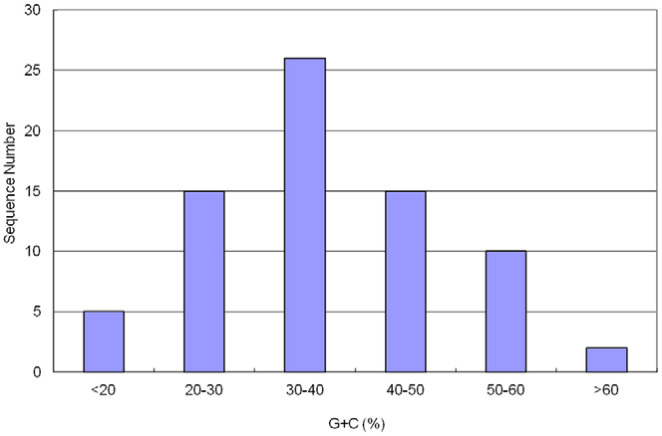
The distribution of GC content. The range is 5.77–61.33% with an average of 37.05±11.78%.

**Figure 6:**
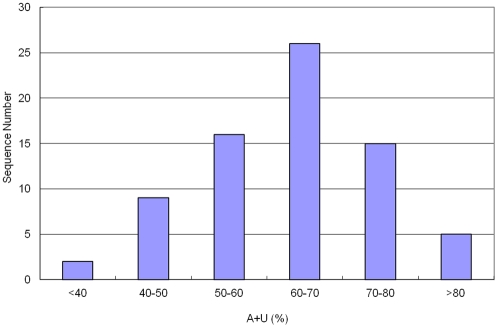
The distribution of AU content. The range is 38.67–94.23% with an average of 62.95±11.78%.

Nucleotides G and C contributes to the formation and stabilization of the secondary structure of stem-loop hairpins because G and C will form three hydrogen bonds with each other but A and U only form two hydrogen bonds with each other. Generally speaking, the more GC content a sequence contains, the more stable the secondary structure of a specific RNA will be. In the identified Asiatic cotton pre-miRNAs, the GC content (37.1±11.8%) was much lower than the AU content (63.0±11.8%) (Table 2, Figures 5 and 6). More than half of the total number of nucleotides were A or U. In this study, we also calculated the A/U and C/G ratio; the ratios of A/U and C/G were 0.94 and 0.76, respectively. This suggests that more of the nucleotide G existed in the pre-miRNA sequences than C however the number of A and U nucleotiudes was almost equal.

**Figure 7:**
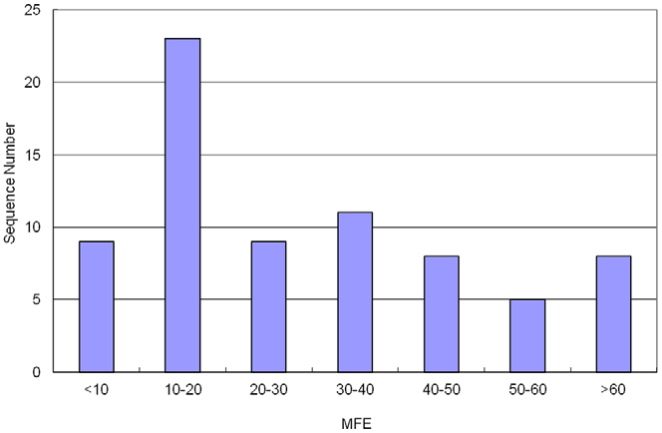
The distribution of MFE. The range of MFE is -3.2 - -232.2 kcal/mol with an average of –35.19±35.12 kcal/mol.

**Figure 8:**
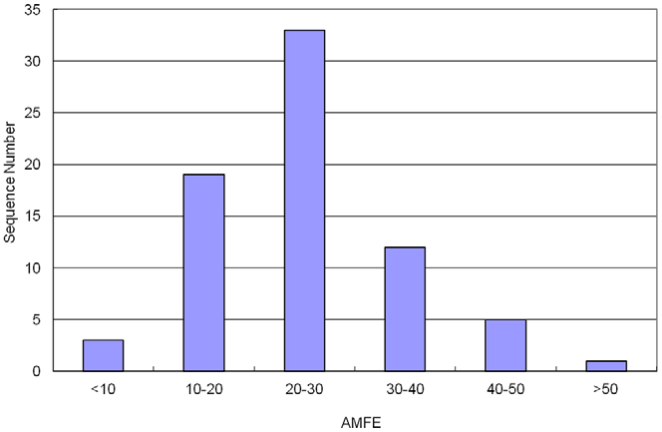
The distribution of AMFE. The range of AMFE is -6.15 - -51.86 kcal/mol with an average of - 24.92±9.21 kcal/mol.

One criterion for measuring the stability of a RNA or DNA secondary structure is the minimal folding free energy (MFE). Usually, the lower the MFE, the more stable the RNA or DNA molecule. The MFE of the 73 identified Asiatic cotton pre-miRNAs varied from -3.2 to -232.2 kcal/mol with an average of -35.19±35.12 kcal/mol (Figure 7). For a majority of Asiatic cotton pre-miRNAs, their MFE are ranged from -10 to -30 kcal/mol. One reason causing the large variance in MFE is the significance in their length. It is also an issue for determining the stability of a RNA or DNA using MFE because different RNA or DNA strands contains a different number of nucleotides. To better measure the stability of RNA or DNA strands, the adjusted minimal folding free energy (AMFE) strategy was developed, which is the MFE of a RNA/DNA sequence that is 100 nt in length. The AMFE of the 73 identified Asiatic cotton pre-miRNAs ranged from - 6.15 to -51.86 kcal/mol with an average of - 24.92 ±9.21 kcal/mol, which is a smaller range as compared with the MFE range. About half of the pre-miRNAs have AMFE values with –20 to –30 kcal/mol (Figure 8).

**Figure 9:**
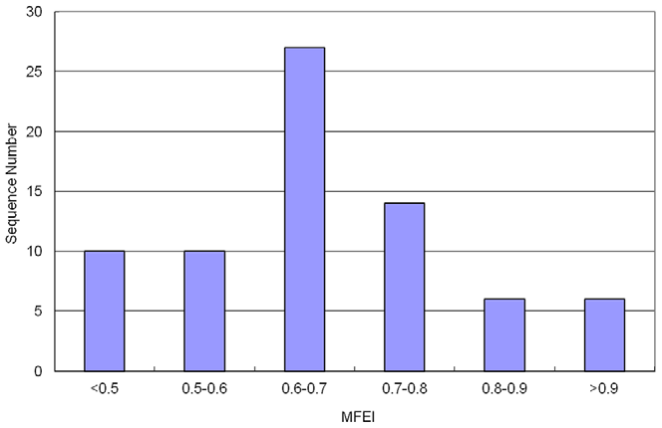
The distribution of MFEI. The range of MFEI is 0.29–1.85 with an average of 0.70±0.25.

The minimal folding free energy index (MFEI) is a new criterion for assaying miRNAs and distinguishing miRNAs from other coding and non-coding RNAs. The MFEI of the identified Asiatic cotton pre-miRNAs ranged from 0.29 to 1.85 with an average of 0.70±0.25 (Figure 9).

### Validation of miRNAs in Asiatic Cotton

Quantitative real-time PCR (qRT-PCR) is a reliable method to determine the expression of specific miRNAs [33]. In this study, we employed qRT-PCR to detect the expression of three predicted miRNAs (miR 156, miR 172 and miR 399). The results show that all of the three predicted miRNAs exist and are highly expressed in Asiatic cotton seedlings.

## Discussion

Although miRNA-related research is one of the hottest research topics in biological or biomedicinal fields [34], [35], miRNA research on cotton is much behind other plant species. Up to now, only several research projects have reported on miRNA identification and expression analysis in cotton [36], [37], [38], [39], [40]. These research projects employed computational approaches as well deep sequencing technology to identify miRNAs from cotton and to investigate their expression profile using qRT-PCR [37] and deep sequencing [40]. However, all of these studies are focused on upland cotton. Based on our best knowledge, no research has been performed on diploid cotton. In this study, our focuswas on an important diploid cotton species, Asiatic cotton, and we were able to successfully identify 73 miRNAs, belonging to 49 families, using an *in silico* comparative genome-based approach. Many of these identified miRNAs play an important role in model plant species. For example, miR 172 controls floral development and phase switch to reproductive growth. miR 399 is a responsive miRNA to environmental stress. We believe these miRNAs also play important roles in Asiatic cotton growth, development, and response to environmental abiotic and biotic stresses.

Newly identified Asiatic cotton miRNAs have similar characteristics to the miRNAs in other plant species [32]. These major parameters include the length of mature miRNAs and miRNA precursors, the nucleotide composition, and the minimal folding free energy (Table 2). This suggests that Asiatic cotton miRNAs have a common ancestor with other plant species and that they share the same regulatory mechanisms.

## Supporting Information

Table S1 **microRNA precursor sequences**
(XLS)
